# Combined value of triglyceride-glucose index and non-high-density lipoprotein cholesterol in predicting early cognitive impairment after acute ischemic stroke

**DOI:** 10.3389/fneur.2026.1713324

**Published:** 2026-03-06

**Authors:** Jingwen Xu, Shanyao Zhu, Ting Wang, Wanxin Lu

**Affiliations:** Department of Internal Medicine-Neurology, The Fourth Affiliated Hospital of Anhui Medical University, Hefei, Anhui, China

**Keywords:** triglyceride-glucose index (TyG index), non-high-density lipoprotein cholesterol (non-HDL-C), acute ischemic stroke (AIS), cognitive impairment, dyslipidemia, diagnostic value, influencing factors

## Abstract

**Background:**

Acute ischemic stroke (AIS) represents a significant global cause of mortality and long-term disability. Cognitive impairment frequently occurs among AIS patients, adversely affecting their functional outcomes. Identifying modifiable risk factors linked to cognitive dysfunction after stroke is thus critical for effective prevention and targeted therapeutic interventions.

**Objective:**

This study explored the relationship between serum triglyceride-glucose (TyG) index and non-high-density lipoprotein cholesterol (non-HDL-C) levels and early cognitive impairment in AIS patients.

**Methods:**

The Neurology Department of Anhui Medical University’s Fourth Affiliated Hospital recruited a total of 235 individuals diagnosed with AIS between September 2023 and January 2025. Patients served as a cognitive impairment group (*n* = 135) and a control group (*n* = 100). Furthermore, participants were dichotomized according to diabetic status, and the predictive value of the TyG index and non-HDL cholesterol for cognitive impairment following acute ischemic stroke was evaluated in these subgroups. The Montreal Cognitive Assessment (MoCA) was used to evaluate cognitive ability at seven days post-stroke; a score below 26 indicated impairment. After identifying independent risk variables for cognitive impairment using logistic regression analysis, the diagnostic value of these factors was determined using receiver operating characteristic (ROC) curve analysis.

**Results:**

The cognitive impairment group exhibited significantly elevated TyG index and non-HDL-C serum levels (*p* < 0.001). Patients with cognitive impairment were older, had less educational attainment, higher NIHSS scores, and reduced MoCA scores (*p* < 0.05). Additionally, glycemic indicators (FPG, HbA1c, TyG index) and lipid markers (TC, non-HDL-C, LDL-C, TG) were markedly elevated, while HDL-C was reduced among cognitively impaired individuals (*p* < 0.05). Patients in the high-TyG group displayed substantially increased glycemic parameters, lipid profiles, and higher diabetes prevalence (*p* < 0.05). Univariate logistic regression revealed each unit rise in TyG index and non-HDL-C (all *p* < 0.05) significantly elevated the risk of cognitive impairment. Both parameters negatively correlated with MoCA scores (both *p* < 0.001). The rise in non-HDL-C levels correlated with the increase in the TyG index (*p* < 0.001), which may indicate that both factors act in a coordinated manner within shared metabolic pathways. The combined predictive model incorporating both TyG index and non-HDL-C exhibited superior diagnostic performance (*p* < 0.001). Regardless of diabetic status, both the TyG index and non-HDL-C demonstrated significant predictive value for post-AIS cognitive impairment. Their combination provided incremental predictive information beyond either marker alone (*p* < 0.001).

**Conclusion:**

Elevated serum levels of TyG index and non-HDL-C independently predict early cognitive impairment in AIS patients, with their combination significantly improving predictive accuracy. These results suggest potential benefits from early metabolic interventions to enhance cognitive recovery post-stroke.

## 1 Introduction

As a predominant factor contributing to global long-term disability and mortality, AIS occurs following an interruption of blood supply to the brain, commonly due to thrombosis or atherosclerotic processes (1). Approximately 30–50% of AIS patients experience different degrees of cognitive impairment, which severely affects their prognosis and quality-of-life (2). A significant and increasingly recognized complication following AIS is the development of early cognitive impairment, which a growing body of evidence now links to systemic metabolic dysregulation, including aberrations in circulating lipid profiles. Although it is increasingly recognized that disordered glucose and lipid metabolism are fundamental to the pathophysiology of this post-stroke cognitive decline (3), the precise metabolic pathways have yet to be fully elucidated.

Insulin resistance (IR) and dyslipidemia are widely recognized as risk factors influencing cognitive impairment after AIS (4–7). The TyG index (8), which integrates IR with lipid metabolism disorders, aggravates cognitive deficits by intensifying neuroinflammatory responses. Meanwhile, non-HDL-C, encompassing all lipoproteins with atherogenic properties, induces endothelial vascular damage and facilitates amyloid-beta (Aβ) deposition (9). Although prior studies have independently linked the TyG index and non-HDL-C to metabolic syndrome, the combined influence of these markers on early cognitive dysfunction following AIS is yet to be fully elucidated (4, 10).

The reliance of current predictive models for cognitive impairment on neuroimaging or complex neuropsychological testing underscores an urgent need for novel and more accessible biomarkers. Therefore, this study was designed to evaluate the TyG index and non-HDL-C as potential markers for early cognitive impairment. We aimed to evaluate their independent and combined predictive power on early cognitive impairment in AIS patients, identifying factors which influenced cognitive impairment in them. This analysis provides a foundation for implementing early clinical interventions targeting these modifiable risk factors. Furthermore, the association with glucose and lipid metabolism disorders suggests that providing potential targets for clinical metabolic intervention holds significant clinical implications. Such approaches are crucial for improving long-term cognitive outcomes in AIS patients.

## 2 Materials and methods

### 2.1 Study design and participants

**Figure 1:**
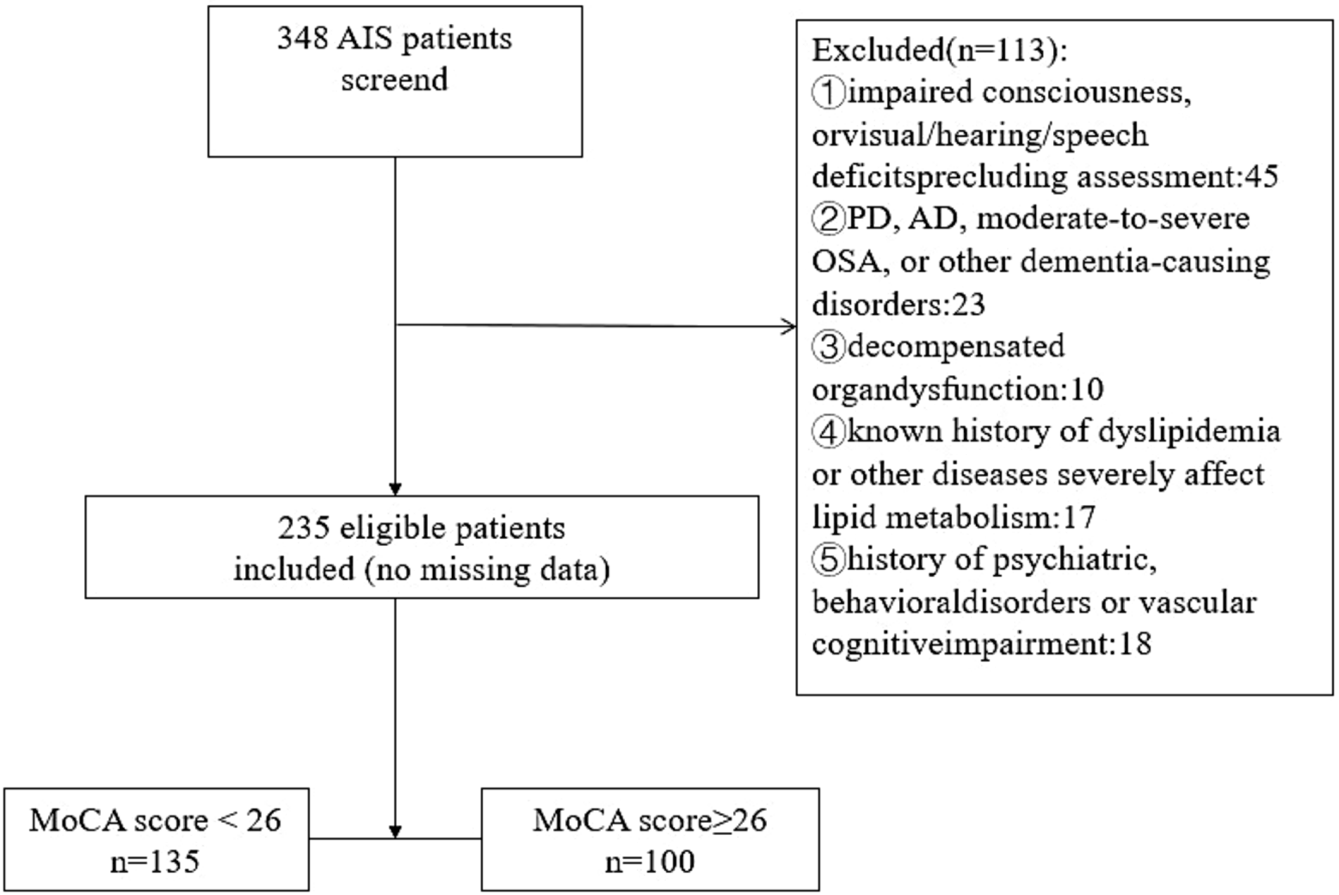
Flow chart of patient recruitment.

Initially, 348 AIS patients were screened by the Neurology Department at Anhui Medical University’s Fourth Affiliated Hospital from September 2023 through January 2025. Following application of specific inclusion and exclusion criteria, 113 patients were removed from the cohort, resulting in a final participant count of 235 (Figure 1). Inclusion criteria were: (1) diagnosis of first-ever ischemic stroke in compliance with the Chinese 2018 AIS guidelines, confirmed by brain imaging (MRI or CT) demonstrating ischemic lesions; (2) hospital admission within seven days from stroke occurrence; (3) age ≥18 years; (4) sufficient cognitive and communicative ability to engage in cognitive testing; and (5) written informed consent provided either by the patient or their legal representative. Exclusion criteria: (1) altered consciousness levels (including delirium) or significant visual, auditory, or speech impairments that may affect the accuracy of cognitive evaluation; (2) previously diagnosed cognitive disorders resulting from neurodegenerative conditions; and (3) diagnosed primary or secondary dyslipidemia (such as familial hypercholesterolemia, nephrotic syndrome, severe hypothyroidism) or systemic diseases significantly affecting lipid metabolism (such as severe liver dysfunction, active inflammatory diseases); (4) decompensated organ dysfunction, with the exception of well-controlled hypertension, coronary artery disease, diabetes or dyslipidemia; and (5) a history of psychiatric disorders or vascular cognitive impairment.

### 2.2 Data collection and clinical assessment

Baseline clinical data, encompassing demographic information, lifestyle habits, medical history, and previous medication use, were gathered within 24 h after hospital admission. Education levels were stratified into two categories: fewer than 12 years of formal education and 12 years or more (Table 1).

**Table 1:** Baseline characteristics by cognitive status in AIS patients.

Demographics	Case group (*n* = 135)	Control group (*n* = 100)	t/Z/x^2^	*P*
Male, *n*(%)	72 (53.5)	55 (55)	0.064	0.800
Age, median (IQR) (years)	71 (66, 75)	63 (56, 69)	−6.741	<0.001
BMI, mean (SD), (kg/m^2^)	24.88 ± 2.28	24.99 ± 2.08	0.381	0.703
Education level, *n* (%)				
Education <12 years	98 (72.6)	60 (60)	4.135	0.042
Education ≥12 years	37 (27.4)	40 (40)		
Smoking	55 (40.7)	47 (47)	0.916	0.338
Drinking	46 (34.1)	45 (45)	2.890	0.089
Medical history, *n* (%)				
Hypertension	93 (68.9)	65 (65)	0.394	0.530
Diabetes mellitus	48 (35.6)	29 (29)	0.347	0.556
Coronary artery disease	26 (19.3)	12 (12)	2.233	0.135
Atrial fibrillation	9 (6.7)	4 (4)	0.782	0.377
Dyslipidemia	21 (15.6)	16 (16)	0.009	0.926
Carotid plaque	93 (68.9)	49 (49)	9.503	0.002
Antihypertensive use	86 (63.7)	62 (62)	0.072	0.789
Antidiabetic use	19 (14.1)	13 (13)	0.056	0.812
Lipid-lowering drug use	8 (5.9)	6 (6)	0.122	0.981
Anti-ischemic drug use	16 (11.9)	7 (7)	1.532	0.216
Clinical characteristics				
NIHSS on admission	5 (3, 7)	3 (2, 4)	−6.247	<0.001
TOAST classification, *n*(%)				
LAA	79 (58.5)	59 (59)	0.543	0.949
Small-vessel occlusion	47 (34.8)	35 (35)		
Cardioembolism	6 (4.4)	3 (3)		
Other/undetermined	3 (2.2)	3 (3)		
Infarct location, *n*(%)				
Critical regions	85 (63.0)	55 (55)	1.513	0.219
Non-critical regions	50 (37.0)	45 (45)		
Laterality, *n*(%)				
Left	77 (57)	55 (55)	0.946	0.623
Right	51 (37.8)	42 (42)		
Bilateral	7 (5.2)	3 (3)		
Modified Fazekas scale, *n*(%)				
Grade 1	42 (31.1)	32 (32)	2.607	0.272
Grade 2	61 (45.2)	45 (45)		
Grade 3	32 (23.7)	23 (23)		
Laboratory characteristics				
FPG, median (IQR) (mmol/L)	6.10 (5.40, 6.90)	5.10 (4.50, 5.70)	−5.849	<0.001
HbA1c, median (IQR) (%)	5.70 (5.20, 6.30)	5.40 (5.20, 6.08)	−2.066	<0.001
Uric acid, median (IQR) (μmol/ L)	321 (278, 366)	343 (298, 395)	−2.333	0.020
Hcy, median (IQR) (μmol/ L)	13.1 (11.5, 14.4)	12.3 (10.4, 14.1)	−2.009	0.045
TC, median (IQR) (mmol/L)	4.33 (3.82, 4.78)	3.97 (3.77, 4.44)	−3.243	0.001
HDL-C, mean (SD) (mmol/L)	1.01 ± 0.15	1.09 ± 0.17	4.170	<0.001
TG, mean (SD) (mmol/L)	1.47 ± 0.47	1.31 ± 0.32	−3.042	0.003
TyG, mean (SD)	8.84 ± 0.44	8.59 ± 0.34	−4.808	<0.001
non-HDL-C, median (IQR) (mmol/L)	3.31 (2.87, 3.76)	2.96 (2.73, 3.28)	−4.662	<0.001
LDL, median (IQR) (mmol/L)	2.80 (2.33, 3.35)	2.65 (2.33, 2.89)	−2.533	0.011
MoCA score, median (IQR)	22 (20, 24)	27 (27, 28)	−13.184	<0.001

Stroke subtyping was performed using criteria from the Acute Stroke Treatment (TOAST) classification (11). Stroke severity was evaluated via the NIH Stroke Scale (NIHSS) (12). Brain MRI imaging, performed within 72 h post-admission, identified infarction locations (left hemisphere, right hemisphere, bilateral involvement) and differentiated between critical regions (frontal and temporal lobes, thalamus, basal ganglia) and non-critical areas (parieto-occipital lobes, cerebellum, brainstem). White matter hyperintensity was rated using the modified Fazekas scale (0 to 3 points), while carotid plaque burden was determined through ultrasonographic examinations. After overnight fasting for at least eight hours, blood samples were drawn the subsequent morning to measure biochemical markers. Cognitive status was evaluated using the MoCA on day 7 post-stroke; scores below 26 indicated cognitive impairment. To reduce education-related biases, two additional points were assigned to individuals unable to read or write, and one extra point was given to those with less than 12 years of formal education (13). TyG index and non-HDL-C levels were calculated using Equations 1, 2, respectively.


TyGindex=ln[TG×FPG/2] (1)

Non−HDL−C=TC−HDL−C (2)

These two indicators have been applied in prior cardiovascular and cognitive research fields (14, 15).

### 2.3 Statistical analysis

The statistical analysis was carried out using SPSS software, specifically version 26.0. The results were analyzed using t-tests or one-way analysis of variance (ANOVA) when suitable, and they were summarized as means ± standard deviations (SD). Medians and interquartile ranges [M (P25, P75)] were given for data that did not follow normal distributions. The Kruskal-Wallis *H* test or the Mann–Whitney *U* test were used to compare two or more groups, respectively. For categorical data, percentages were computed, and the chi-square (χ^2^) test was used to look for differences between groups. To determine characteristics that independently predict cognitive impairment following AIS, logistic regression models were built. To measure prediction accuracy, ROC curves with matching AUC values were developed. A *p*-value less than 0.05 was considered statistically significant.

## 3 Results

### 3.1 Cognitive impairment vs. intact cognition in early AIS

Out of the enrolled participants, early cognitive impairment was identified in 135 (57%) AIS patients. Comparative analysis (Table 1) demonstrated significant differences between cognitively impaired and cognitively intact groups: patients with cognitive deficits were older, had fewer years of education, higher admission NIHSS scores, and lower MoCA scores (all *p* < 0.005).

Glycemic indices (FPG, HbA1c, TyG index; all *p* < 0.001) were significantly elevated among cognitively impaired patients. Significant differences were also observed concerning lipid metabolic parameters. Specifically, increased occurrence of carotid plaques (*p* = 0.002), higher TC, TG, LDL-C, and non-HDL-C concentrations (all *p* < 0.05), and decreased HDL-C concentration (*p* < 0.001) were identified. In addition, cognitively impaired subjects exhibited significantly raised UA and HCY (all *p* < 0.05) serum concentrations.

### 3.2 Logistic regression analysis of risk factors for early cognitive impairment in AIS

To tackle the issue of multicollinearity, following multivariate analyses only included covariates that had substantial univariate relationships with cognitive impairment. To adjust for the confounding effects of lipid-lowering and antidiabetic medications, we incorporated the use of these agents as covariates into the multivariable logistic regression analysis (Table 2). The cognitive impairment risk significantly increased with each unit rise in TyG index and non-HDL-C, according to the univariate logistic regression results (all *p* < 0.005). Cognitive impairment was independently predicted by having an older age, a shorter duration of education, higher NIHSS scores at admission, raised fasting phosphorus and triglyceride levels, higher levels of LDL-C, and lower levels of HDL-C (all *p* < 0.005).

**Table 2:** Logistic regression analysis of risk factors for cognitive impairment in AIS patients.

Demographics	Univariate analysis OR (95%CI)	*P*	Multivariate analysis OR (95%CI)	*P*
Age (years)	1.126 (1.084, 1.170)	<0.001	1.135 (1.077, 1.196)	<0.001
Male, *n* (%)	0.935 (0.556, 1.572)	0.800		
BMI, (kg/m^2^)	0.977 (0.868, 1.100)	0.702		
Education level, *n* (%)				
Education <12 years	0.566 (0.327, 0.982)	0.043	0.741 (0.507, 1.124)	0.021
Education ≥12 years				
Smoking	0.875 (0.460, 1.306)	0.339		
Drinking	0.732 (0.371, 1.074)	0.090		
Medical history, *n* (%)				
Hypertension	1.192 (0.688, 2.065)	0.530		
Diabetes mellitus	1.196 (0.660, 2.167)	0.556		
Coronary artery disease	1.749 (0.835, 3.664)	0.138		
Atrial fibrillation	1.124 (0.832, 1.548)	0.382		
Dyslipidemia	1.203 (0.687, 1.758)	0.426		
Carotid plaque	1.192 (0.896, 1.634)	0.532		
Antihypertensive use	1.076 (0.630, 1.837)	0.789		
Antidiabetic use	1.096 (0.514, 2.340)	0.812	0.488 (0.286, 1.034)	0.054
Lipid-lowering drug use	0.987 (0.331, 2.940)	0.981	0.674 (0.489, 1.237)	0.358
Anti-ischemic drug use	1.236 (0.706, 2.054)	0.221		
Clinical characteristics				
NIHSS on admission	1.598 (1.355, 1.884)	<0.001	1.427 (1.157, 1.760)	0.001
TOAST classification, *n* (%)				
LAA	1.022 (0.605, 1.726)	0.936		
Small-vessel occlusion	1.037 (0.601, 1.787)	0.897		
Cardioembolism	1.174 (0.728, 1.934)	0.571		
Other/undetermined	0.845 (0.342, 1.630)	0.710		
Infarct location, *n* (%)				
Critical regions	0.719 (0.425, 1.217)	0.219		
Non-critical regions				
Laterality, *n* (%)				
Left	1.004 (0.642, 1.571)	0.985		
Right				
Bilateral				
Modified Fazekas scale, *n* (%)	1.324 (0.935, 1.876)	0.114		
Laboratory characteristics				
FPG (mmol/L)	1.782 (1.400, 2.269)	<0.001	1.583 (1.049, 2.419)	<0.001
HbA1C (%)	1.134 (0.827, 1.678)	0.076		
Uric acid (μmol/L)	1.026 (0.982, 1.350)	0.083		
HCY (μmol/L)	1.082 (0.977, 1.436)	0.062		
TC (mmol/L)	1.120 (0.824, 1.723)	0.073		
HDL-C (mmol/L)	0.576 (0.350, 0.896)	0.020	0.382 (0.011, 0.654)	0.003
TG (mmol/L)	1.352 (1.120, 2.134)	0.011	1.236 (1.078, 2.456)	0.043
LDL-C (mmol/L)	1.280 (1.012, 2.340)	0.032	1.468 (1.233, 2.782)	<0.001
non-HDL-C (mmol/L)	1.367 (1.124, 2.543)	0.009	1.790 (1.425, 3.457)	<0.001
TyG	1.452 (1.176, 2.869)	0.002	2.116 (1.327, 4.652)	0.013

### 3.3 Relationship between TyG index/non-HDL-C and MoCA scores

**Figure 2:**
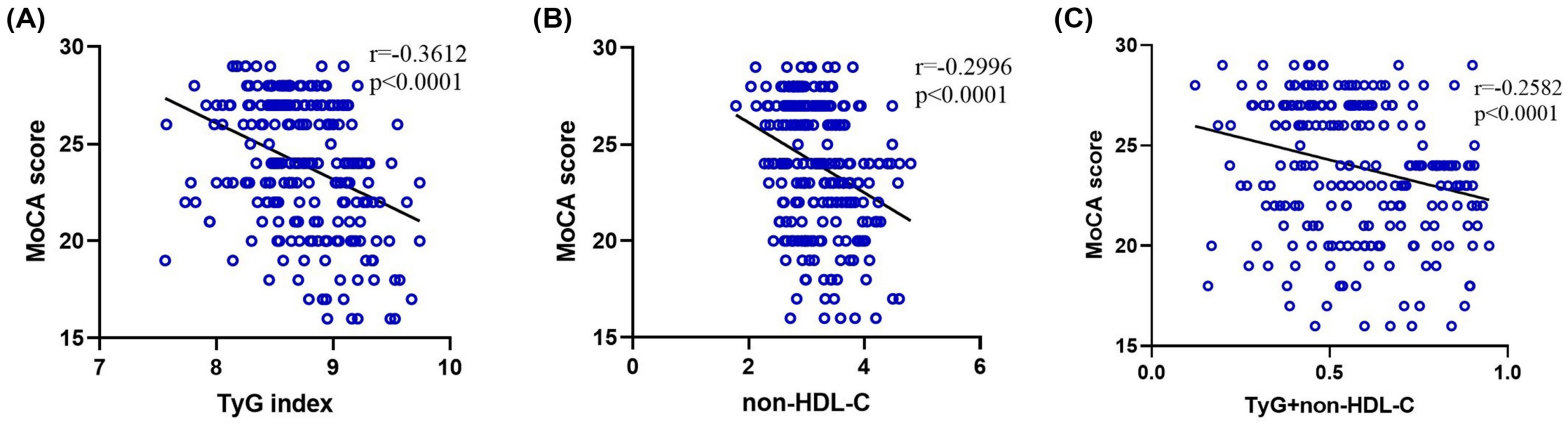
**(A–C)** Association between the TyG index, non-HDL-C, and MoCA score.

**Figure 3:**
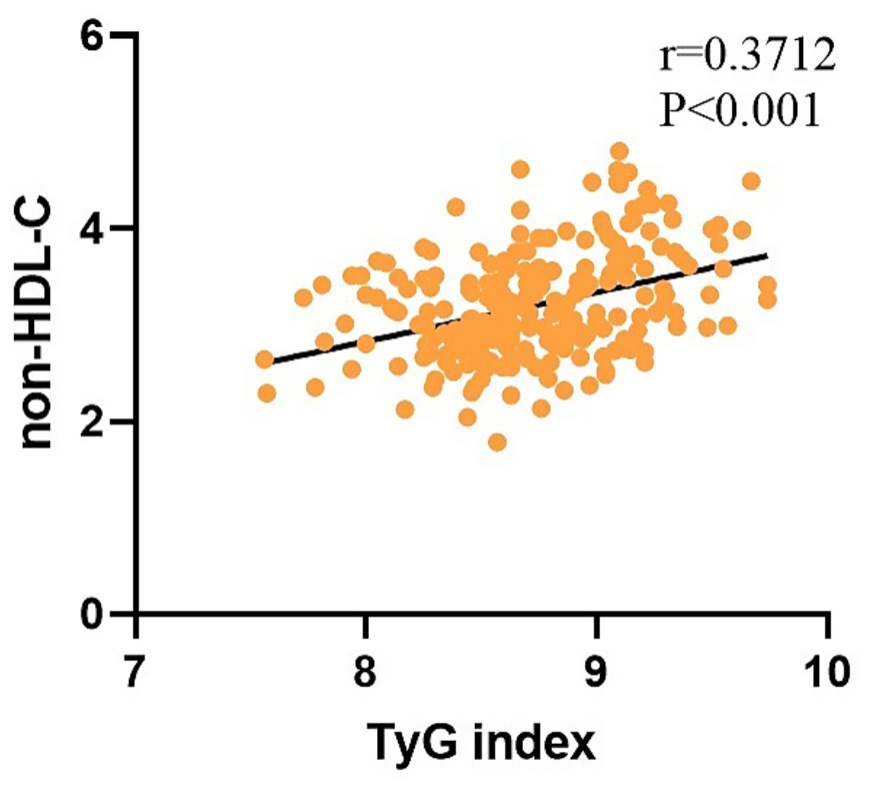
Correlation between TyG index and non-HDL-C.

Both TyG index and non-HDL-C exhibited negative correlations with MoCA scores (Figure 2), confirmed by Spearman correlation analyses (*p* < 0.001). Additionally, the combined TyG and non-HDL-C score maintained a significant negative association with MoCA scores (*p* < 0.001). Furthermore, TyG index was positively associated with increased non-HDL-C levels (*p* < 0.001; Figure 3).

### 3.4 Predictive power of TyG index and non-HDL-C for early cognitive impairment after AIS

**Figure 4:**
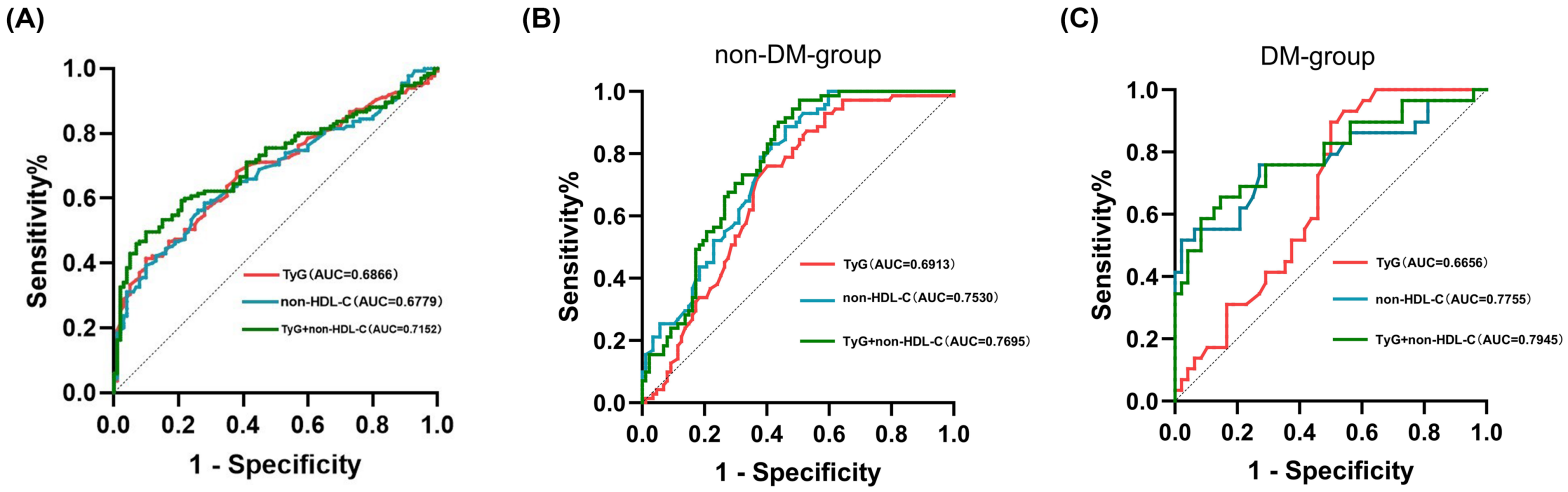
**(A–C)** TyG index and non-HDL-C: individual and combined prediction of early cognitive impairment in AIS.

**Figure 5:**
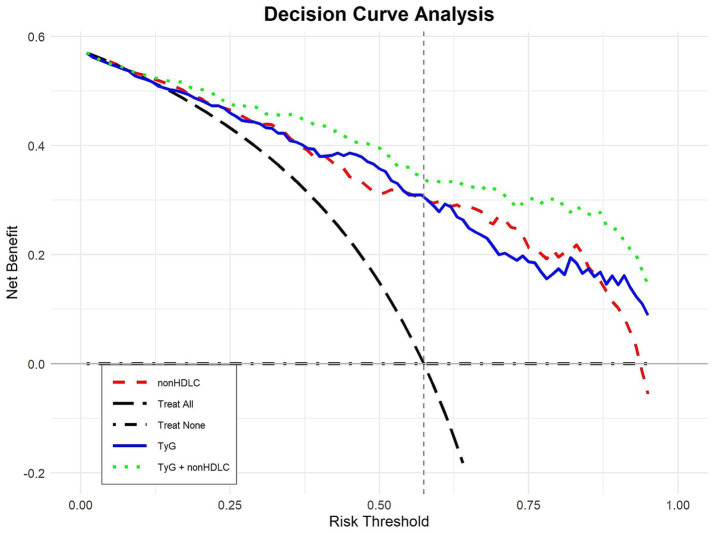
DCA of TyG and non-HDL-C for early cognitive impairment after AIS.

Cognitive dysfunction at an early stage of AIS was predicted using the TyG index and non-HDL-C using the ROC approach. Concurrently, decision curve analysis (DCA) plots were constructed to evaluate the clinical utility of the models. Following analysis, a TyG index cutoff of 9.01 was found to be appropriate, resulting in a sensitivity of 61.5%, specificity of 71%, and an AUC of 0.689 (*p* < 0.001). The optimal diagnostic threshold for non-HDL-C was determined to be 3.28, with specificity and sensitivity values of 66.3 and 63.8%, respectively, and an AUC of 0.678 (*p* < 0.001). The predictive accuracy was further improved when the TyG index and non-HDL-C were combined, with a sensitivity of 66.3%, specificity of 77%, and a greater AUC of 0.715 (*p* < 0.001), outperforming the predictive performance of each biomarker separately (Table 3; Figure 4). The DCA curve analyses further demonstrated that within the clinically relevant risk threshold range of 0.00 to 1.00, the net benefit curve of the model combining the TyG index and non-HDL-C lay consistently above those of the universal screening and no screening strategies, which corroborates that this model can yield a positive clinical net benefit for the risk stratification of early cognitive impairment following AIS (Figure 5).

**Table 3:** TyG index and non-HDL-C for diagnosing early cognitive impairment in AIS.

Indicator	AUC	Sensitivity (%)	Specificity (%)	Optimal cut-off value	*P*	95%CI
TyG	0.689	61.5	71.0	9.01	<0.001	0.620–0.753
non-HDL-C	0.678	63.8	75.0	3.28	<0.001	0.610–0.746
TyG + non-HDL-C	0.715	66.3	77.0	0.66	<0.001	0.650–0.780

### 3.5 Predictive value of TyG index and non-HDL-C for early cognitive impairment after AIS according to the presence or absence of diabetes

Subgroup analyses were performed stratified by diabetes status among enrolled participants. Within the diabetes mellitus (DM) subgroup, the TyG index and non-HDL-C yielded optimal cut-off values of 9.11 (AUC = 0.667, *p* = 0.013) and 2.91 (AUC = 0.776, *p* < 0.001), respectively. Notably, the combined model incorporating both the TyG index and non-HDL-C achieved an elevated AUC of 0.795 (*p* < 0.001), with a sensitivity of 76.4% and specificity of 71.5%. In the non-diabetes mellitus (non-DM) subgroup, both the TyG index (AUC = 0.691, *p* < 0.001; optimal cut-off = 8.64) and non-HDL-C (AUC = 0.753, *p* < 0.001; optimal cut-off = 3.12) demonstrated robust predictive efficacy. The combined model again outperformed each biomarker individually, yielding an AUC of 0.770 (*p* < 0.001), with 69.7% sensitivity and 79.2% specificity (Table 4; Figure 4).

**Table 4:** Value of TyG index and non-HDL-C for diagnosing early cognitive impairment in AIS according to the presence or absence of diabetes.

Indicator	AUC	Sensitivity (%)	Specificity (%)	Optimal cut-off value	*P*	95%CI
DM-group						
TyG	0.667	65.8	77.6	9.11	0.013	0.547–0.785
Non-HDL-C	0.776	74.7	70.8	2.91	<0.001	0.658–0.893
TyG + non-HDL-C	0.795	76.4	71.5	0.69	<0.001	0.685–0.904
Non-DM-group						
TyG	0.691	61.5	74.1	8.64	<0.001	0.609–0.774
Non-HDL-C	0.753	64.9	72.6	3.12	<0.001	0.679–0.827
TyG + non-HDL-C	0.770	69.7	79.2	0.57	<0.001	0.697–0.842

## 4 Discussion

Clinically, the TyG index and non-HDL-C serve as well-recognized indicators of IR and lipid abnormalities, respectively (16). Dysregulation in lipid metabolism is closely linked to IR (17), highlighting a shared pathological mechanism between these two biomarkers, characterized by disturbed glucose and lipid homeostasis. Emerging evidence suggests elevated TyG index levels correlate with PSCI in AIS patients, and implies that reductions in TG and FPG might offer protective benefits (14, 18). This aligns with our finding of an inverse correlation between TyG index and MoCA scores. Besides, elevated non-HDL-C may promote vascular inflammation and disruption of the blood–brain barrier, indirectly impacting cognition (9, 19), further supporting its role in early AIS cognitive impairment.

When it comes to abnormalities of lipid or glucose metabolism, previous research has mostly focused on non-HDL-C and the TyG index separately. Nevertheless, there is a lack of information indicating the combined predictive relevance of these factors in early cognitive impairment following AIS. For that reason, we set out to fill this knowledge vacuum by investigating whether or not serum non-HDL-C and TyG index could predict cognitive decline in patients with AIS. The present results provide credence to the idea that the TyG index and non-HDL-C both stand on their own as potential indicators of early cognitive impairment in AIS patients. Furthermore, their joint assessment enhances predictive power, a novel finding not extensively documented in previous studies.

Elevated blood glucose levels typically reflect insufficient insulin secretion or diminished insulin responsiveness, while hypertriglyceridemia is a hallmark characteristic of insulin resistance-related lipid abnormalities. In line with previous evidence (20), our study revealed a significant positive correlation between non-HDL-C and TyG index. We hypothesize that insulin resistance is the key pathophysiological link. An elevated TyG index denotes a more severe insulin-resistant state, which is a major contributor to atherogenic dyslipidemia. In this state, impaired glucose disposal and accelerated lipolysis generate a surplus of substrates that fuel hepatic triglyceride synthesis (21). Furthermore, impaired insulin signaling related to hepatic triglyceride and very-low-density lipoprotein (VLDL) metabolism contributes to the excessive release of triglyceride-rich VLDL particles into circulation. Given that VLDL and its remnant particles constitute a substantial portion of non-HDL-C and subsequently transform into remnant lipoproteins and LDL, increased levels of non-HDL-C frequently arise under such metabolic conditions (22).

Importantly, our study demonstrated that the combined use of the TyG index and non-HDL-C had superior synergistic predictive power (AUC = 0.715) compared with either marker alone or with traditional combinations. A plausible explanation for our findings is a “dual-hit” model targeting the integrity of the neurovascular unit (NVU), the pathological cornerstone of early post-AIS cognitive impairment (23). The first “hit” is metabolic, driven by insulin resistance: impaired cerebral glucose uptake and utilization create an energy deficit that compromises neuronal function, while concomitant hyperglycemia increases oxidative stress and damages both neurons and vascular endothelial cells (24). The second “hit” is vascular, precipitated by elevated non-HDL-C: this dyslipidemia promotes vascular pathology that culminates in cerebral small vessel disease (CSVD), forming the structural basis for cognitive deficits (25). This interaction creates a vicious cycle: compromised blood vessels fail to provide compensatory blood flow to energy-starved neurons, while these metabolically compromised neurons have reduced tolerance to ischemia and hypoxia. We propose that insulin resistance, as indicated by the TyG index, produces the primary metabolic insult, compromising neuronal and glial function and lowering the threshold for ischemic damage. Concurrently, elevated non-HDL-C imposes a structural burden by compromising vascular architecture and blood–brain barrier integrity, thereby deteriorating the neuronal microenvironment (26). This synergistic crosstalk ultimately precipitates endothelial dysfunction, which is manifested by blood–brain barrier disruption, amyloid-*β* accumulation, and pro-inflammatory cytokine release (27), and results in cognitive impairment that is more severe than the sum of the individual effects of each factor.

Furthermore, subgroup analyses stratified by diabetes mellitus (DM) status were conducted in this study, revealing divergent predictive performance of the TyG index and non-HDL-C between the diabetic and non-diabetic subgroups. In the DM subgroup, non-HDL-C outperformed the TyG index in discriminative capacity. This observation is ascribable to the distinctive pathophysiological traits of diabetic patients: chronic hyperglycemia is frequently accompanied by secondary dyslipidemia in this population, and non-HDL-C, as a core marker of dyslipidemia, directly accelerates the progression of CSVD and neuronal injury. These two pathological alterations constitute the pivotal pathological underpinnings of early cognitive impairment following AIS. Of note, the combined model integrating both indices still attained the most favorable predictive efficacy (AUC = 0.795), suggesting that the synergistic interplay between the metabolism-related TyG index and lipid profile-associated non-HDL-C remains complementary and enhances predictive precision, even among diabetic patients with baseline metabolic disturbances. In the non-diabetic subgroup, both the TyG index and non-HDL-C showed robust predictive utility, with no substantial disparity in their standalone predictive strength. We postulate that IR and dyslipidemia act as relatively independent risk factors for post-AIS cognitive impairment in individuals without DM, with neither factor playing a predominant role in the pathological cascade. These two perturbations damage the NVU through discrete pathways: IR incurs metabolic energy insufficiency, whereas elevated non-HDL-C leads to structural vascular deterioration, a mechanism that aligns closely with the dual-hit model proposed earlier in this work. Within this non-diabetic subgroup, the combined model likewise achieved superior predictive performance relative to either biomarker alone (AUC = 0.770). This finding corroborates that the synergistic crosstalk between metabolic and vascular factors is conserved across different diabetic statuses, further justifying the clinical value of the combined application of these two simple, non-invasive indicators.

Our findings highlight the interconnected roles of the TyG index and non-HDL-C in the complex pathophysiology of cognitive impairment post-AIS. This indicates that clinical interventions can be targeted at these factors, such as intensifying lipid-modifying drug therapy for patients with dyslipidemia and stabilizing blood glucose as early as possible for those with glucose metabolism disorders. These measures are expected to reduce the incidence of early cognitive deficits and improve patient prognosis.

Several limitations should be acknowledged in interpreting this research. Firstly, this is a single-center study with a relatively small sample size, which may restrict the generalizability of our results and preclude the complete exclusion of potential selection bias. Second, although we recorded the use of lipid-modifying agents, the potential confounding effects of these medications, such as statins, ezetimibe, and PCSK9 inhibitors, were not adjusted for in our analyses. Third, our study lacked key inflammatory biomarkers (e.g., C-reactive protein) and did not include mechanistic biomarkers such as measures of inflammation or amyloid-*β* (Aβ), which warrants further investigation. Fourth, we only conducted a single MoCA assessment at 7 days post-stroke onset and did not perform follow-up cognitive evaluations at the conventional 3–6 months post-stroke time point; additionally, domain-specific and continuous MoCA score analyses were not carried out, which may restrict the comprehensive characterization of acute-phase cognitive impairment and the precise exploration of its association with the TyG index and non-HDL-C. Future multicenter studies with larger sample sizes are thus warranted to validate our findings, and such investigations should incorporate long-term cognitive follow-up assessments, domain-specific and continuous MoCA analyses, as well as the detection of inflammatory and mechanistic biomarkers. Integrating radiomics and metabolomics into subsequent research may also help to further elucidate the potential pathological pathways underlying acute post-AIS cognitive impairment.

## Data Availability

The raw data supporting the conclusions of this article will be made available by the authors, without undue reservation.
